# A Prospective, Randomized Comparative Study of Intravenous and Perineural Dexamethasone as an Adjuvant to Levobupivacaine in Ultrasound-Guided Supraclavicular Brachial Plexus Block for Adult Patients Undergoing Elective Upper Limb Surgery

**DOI:** 10.7759/cureus.103753

**Published:** 2026-02-17

**Authors:** Rashmi Soni, Sweta Singh, Sudhir K Dhama, Pramod Chand

**Affiliations:** 1 Anaesthesiology, Critical Care and Pain Medicine, Lala Lajpat Rai Memorial Medical College, Meerut, IND

**Keywords:** dexamethasone, levobupivacaine, perineural vs intravenous, supraclavicular brachial plexus block, ultrasound-guided regional anaesthesia

## Abstract

Background

Ultrasound-guided supraclavicular brachial plexus block is a widely used regional anesthesia technique for upper limb surgeries, offering effective intraoperative and postoperative analgesia. Dexamethasone, a corticosteroid with analgesic and anti-inflammatory properties, is often used as an adjuvant via either the perineural or the intravenous route to prolong block duration. This study aimed to compare the efficacy of intravenous versus perineural dexamethasone as adjuvants to levobupivacaine in ultrasound-guided supraclavicular blocks.

Methods

A prospective, randomized, double-blind study was conducted at Sardar Vallabh Bhai Patel Hospital, Meerut, over 18 months. Sixty American Society of Anesthesiologists (ASA) I/II adult patients aged 18-60 years undergoing elective upper limb surgeries were randomly allocated into two equal groups. Group A received 19 mL of 0.5% levobupivacaine with 1 mL of normal saline perineurally and 4 mg (1 mL) of intravenous dexamethasone. Group B received 19 mL of 0.5% levobupivacaine with 4 mg (1 mL) of perineural dexamethasone and 1 mL of intravenous saline. Block performance and drug preparation were done under sterile conditions using ultrasound guidance. Primary outcomes included onset and duration of sensory and motor block. Secondary outcomes were postoperative analgesia duration, hemodynamic changes, pain scores, and complications.

Results

Perineural dexamethasone (Group B) resulted in significantly faster onset and longer duration of sensory and motor block compared to intravenous administration (Group A) (p < 0.05). The duration of postoperative analgesia was also significantly prolonged in Group B. Hemodynamic parameters remained stable in both groups, and no serious complications were observed.

Conclusion

Perineural dexamethasone is more effective than intravenous dexamethasone in enhancing the onset and duration of levobupivacaine-induced supraclavicular brachial plexus block. It also provides superior postoperative analgesia without increasing adverse effects. It is recommended as the preferred route for dexamethasone administration in upper limb surgeries.

## Introduction

Since Halstead introduced the first brachial plexus block using cocaine in 1884, the technique has significantly evolved from blind approaches to nerve stimulator-assisted and ultrasound-guided methods [1-4]. Brachial plexus blocks now serve as a reliable alternative to general anesthesia for upper limb surgeries, providing excellent perioperative analgesia, reducing hospital stays, lowering costs, and minimizing the side effects of general anesthesia [4-6].

Of the various approaches--interscalene, supraclavicular, infraclavicular, and axillary--the supraclavicular technique is widely preferred for surgeries at or below the elbow [7,8]. Often referred to as the “spinal of the arm,” it offers dense anesthesia with rapid onset and requires a smaller volume of anesthetic. It may be used alone or as an adjunct to general anesthesia to enhance perioperative pain control [9-12].

Ultrasound guidance has further improved the efficacy and safety of supraclavicular blocks, offering real-time visualization, precise drug deposition, faster onset, reduced anesthetic volume, and fewer complications such as pneumothorax. The use of echogenic needles enhances safety and accuracy [13-15].

To prolong the duration and quality of block, adjuvants like alpha-2 agonists (clonidine, dexmedetomidine), opioids (fentanyl, tramadol), and corticosteroids (dexamethasone) are often used. Levobupivacaine, a pure S(-)-enantiomer of bupivacaine, is preferred due to its favorable safety profile, particularly lower cardiotoxicity [8,16,17].

Dexamethasone, a synthetic corticosteroid with anti-inflammatory, analgesic, and antiemetic properties, is used via perineural or intravenous routes to enhance block duration. It may act via vasoconstriction, direct nerve membrane effects, and activation of inhibitory potassium channels [9,17-19]. Perineural dexamethasone has been shown to shorten the onset and prolong the duration of both sensory and motor blocks, as well as analgesia, with minimal adverse effects. Intravenous dexamethasone is also known to reduce postoperative pain, nausea, and vomiting [20]. The present study aims to compare the effects of intravenous versus perineural dexamethasone as an adjuvant to levobupivacaine in ultrasound-guided supraclavicular blocks for adult patients undergoing elective upper limb surgeries.

## Materials and methods

Study design and setting

This was a prospective, randomized, double-blind, parallel-group, controlled study conducted at Sardar Vallabhbhai Patel Hospital affiliated with Lala Lajpat Rai Memorial Medical College, Meerut. The study duration was 18 months, from May 2023 to November 2024. Ethical clearance was obtained from the institutional ethics committee (SC-1-2024/4486), and all participants provided written informed consent prior to enrolment. The study is also registered with the Clinical Trials registry of India (CTRI/2025/02/080286).

Participants

**Figure 1:**
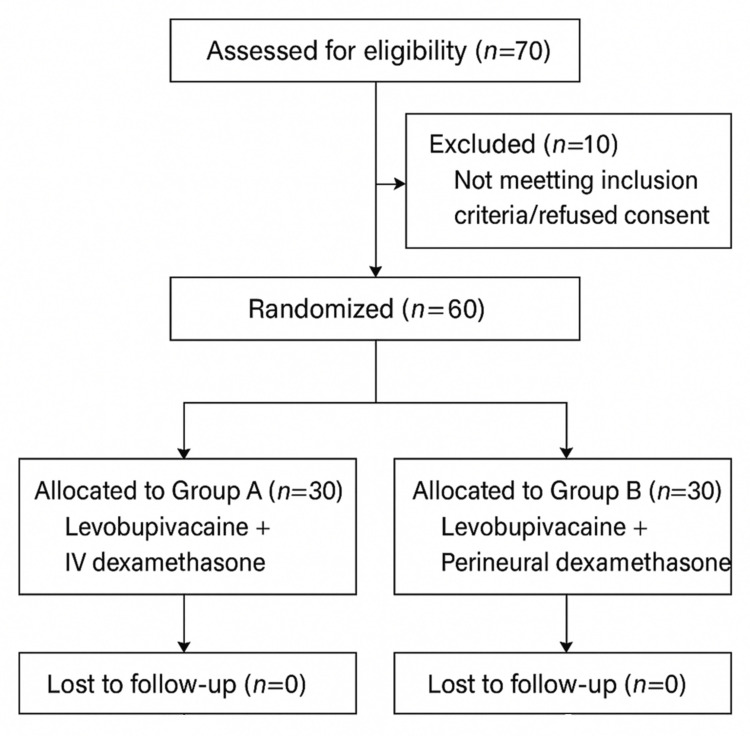
CONSORT (Consolidated Standards of Reporting Trials) 2010 flow diagram depicting patient enrollment, randomization, allocation, follow-up, and analysis

Eligible participants were adults aged 18 to 60 years, of either sex, classified as American Society of Anaesthesiologists (ASA) physical status I or II [21], and scheduled for elective upper limb surgery under ultrasound-guided supraclavicular brachial plexus block (Figure 1). Inclusion also required a body mass index (BMI) between 18 and 24 kg/m². Exclusion criteria included refusal to participate, local infection at the injection site, coagulopathy, pregnancy, first-to-third degree heart block, peripheral neuropathy, or known allergy to local anesthetic agents.

Interventions

Patients were randomly assigned to one of two groups (n=30 each).

Group A (IV Dexamethasone Group)

Patients received 19 mL of 0.5% levobupivacaine with 1 mL of normal saline (total 20 mL) for a supraclavicular block, and 4 mg (1 mL) dexamethasone was administered intravenously.

Group B (Perineural Dexamethasone Group)

Patients received 19 mL of 0.5% levobupivacaine with 4 mg (1 mL) dexamethasone (total 20 mL) for the block, and 1 mL of intravenous normal saline.

Drug preparation was done by an independent anesthesiologist not involved in patient care or data collection. All blocks were performed under ultrasound guidance using standard sterile technique.

Outcomes

The primary outcomes were onset time and duration of sensory and motor block. The secondary outcomes included duration of postoperative analgesia (time to first rescue analgesic), hemodynamic changes (heart rate, mean arterial pressure, systolic and diastolic blood pressure), pain scores using the numeric rating scale (NRS) [22] and incidence of complications (e.g., drowsiness, nausea, vomiting, Horner’s syndrome, phrenic nerve palsy, pneumothorax, and signs of local anesthetic toxicity).

Block onset and duration were assessed using the pin-prick method [23] for sensory block (3-point scale) and the Modified Bromage scale [24] for motor block. Pain was assessed using NRS at 12 and 24-hour intervals postoperatively.

Sample size

A total of 60 participants were included, with 30 in each group. The sample size was based on previously published studies and calculated to achieve adequate precision of 95% confidence limits for the primary outcome variables.

Randomization and blinding

Randomization was performed using a computer-generated random number table. Allocation was concealed in sealed opaque envelopes. The anesthesiologist performing the block and the patient were both blinded to group allocation. A separate anesthesiologist not involved in data collection prepared the study drugs.

Data collection and monitoring

Baseline clinical data, vitals, and preoperative assessment (PAC) findings were recorded. Standard intraoperative and postoperative monitoring was conducted at specific time intervals: 0, 5, 10, 15, 30, 60, 90, 120, 150, and 180 minutes, and at 6, 12, and 24 hours. Complications, if any, were recorded and managed accordingly.

Statistical analysis

Data were entered in Microsoft Excel. Statistical analysis was performed using IBM SPSS Statistics for Windows, Version 24.0 (IBM Corp., Armonk, New York, USA). Descriptive statistics, such as mean, standard deviation, median (IQR), frequencies, and percentages, were used. Independent t-tests or chi-square tests were applied for between-group comparisons depending on the data type. A p-value of < 0.05 was considered statistically significant.

## Results

There were no significant differences between Groups A and B in age (p=0.648), sex (p=1.000), BMI (p=0.818), or ASA grade (p=1.000), indicating well-matched baseline characteristics (Table 1).

**Table 1:** Comparison of baseline characteristics between groups Values are presented as mean ± standard deviation or number (%). BMI: body mass index; ASA: American Society of Anaesthesiologists p-values were calculated using the Independent t-test for continuous variables and the chi-square test for categorical variables. “–” indicates not applicable. A p-value of <0.05 is considered significant.

Baseline Characteristics		Groups		t value	p-value
		A	B		
Age (years)		38.17±12.50	39.67±12.82	-.459	.648
Sex	Male	17 (56.7%)	17 (56.7%)	-	1.000
	Female	13 (43.3%)	13 (43.3%)		
BMI (kg/m^2^)		21.15±2.12	21.27±1.78	-.231	.818
ASA	Grade 1	17 (56.7%)	17 (56.7%)	-	1.000
	Grade 2	13 (43.3%)	13 (43.3%)		

The study found a comparable surgery duration (p=0.967), but Group B had significantly slower onset and prolonged duration of sensory and motor blocks, and longer analgesia (all p=0.001) (Table 2). The heart rate remained comparable between Groups A and B at all measured time intervals from baseline to 24 hours postoperatively, with no statistically significant differences (all p > 0.05) (Table 3).

**Table 2:** Comparison of duration of surgery, sensory and motor block characteristics, and duration of analgesia between Groups A and B Values are presented as mean ± standard deviation. min: minutes p-values were calculated using the independent t-test. A p-value of <0.05 is considered significant.

Parameters	Groups		t value	p-value
	A	B		
Duration of surgery	98.17±32.73	98.50±30.18	-.041	.967
Sensory block onset	10.37±1.97	20.13±3.09	-14.58	.001
Sensory block duration	405.33±39.28	866.33±82.73	-27.57	.001
Motor block onset	12.60±2.50	22.27±2.96	-13.67	.001
Motor block duration	381.67±38.24	1060.00±96.13	-35.91	.001
Duration of analgesia	470.0±46.24	1171.67±96.74	-35.84	.001

**Table 3:** Comparison of heart rate (beats per minute) at different time intervals between Groups A and B Values are presented as mean ± standard deviation. HR: heart rate; bpm: beats per minute p-values were calculated using the independent t-test. A p-value of <0.05 is considered significant.

Heart Rate (Beats per minute)	Groups		t value	p-value
	A	B		
Baseline	82.07±12.98	83.20±11.63	-.356	.723
0 min	86.07±12.13	86.73±11.61	-.217	.829
5 min	88.93±12.15	89.13±11.40	-.066	.948
10 min	89.60±11.67	92.07±10.14	-.874	.386
15 min	87.77±11.54	90.60±9.86	-1.02	.311
30 min	85.67±10.90	89.33±9.43	-1.39	.169
60 min	83.93±11.68	87.60±9.44	-1.34	.186
90 min	83.20±10.73	85.93±9.46	-1.05	.300
120 min	80.43±10.88	83.20±8.94	-1.08	.286
150 min	80.67±10.70	80.93±8.53	-.107	.915
180 min	79.53±10.58	78.53±8.85	.397	.693
6 hours	76.73±10.74	76.07±8.94	.261	.795
12 hours	75.20±11.05	75.20±8.84	.000	1.00
24 hours	74.27±11.27	74.40±7.76	-.053	.958

The comparison of systolic blood pressure (SBP) between Groups A and B across multiple time intervals showed no statistically significant differences at any time point (p > 0.05). Both groups had nearly identical baseline SBP (124.67±9.18 vs. 124.87±7.89 mmHg; p = 0.928). Slightly higher SBP values were observed in Group A at 30, 60, and 90 minutes, with p-values approaching significance (0.058, 0.097, and 0.070, respectively), suggesting a trend but not reaching statistical significance. Overall, SBP remained comparable between the two groups throughout the 24-hour period (Table 4).

**Table 4:** Comparison of systolic blood pressure (mmHg) at different time intervals between Groups A and B Values are presented as mean ± standard deviation. SBP: systolic blood pressure p-values were calculated using the Independent t-test. A p-value <0.05 is considered significant.

Systolic Blood Pressure (mmHg)	Groups		t value	p-value
	A	B		
Baseline	124.67±9.18	124.87±7.89	-.091	.928
0 min	125.27±8.92	124.87±7.87	.184	.855
5 min	125.33±9.10	124.33±7.52	.464	.644
10 min	125.06±8.97	124.13±7.06	.448	.656
15 min	125.13±8.33	123.13±6.84	1.02	.314
30 min	124.67±8.16	121.07±6.10	1.94	.058
60 min	124.07±6.99	121.20±6.14	1.69	.097
90 min	123.13±6.82	120.07±6.02	1.85	.070
120 min	121.93±7.67	119.93±6.02	1.12	.266
150 min	120.27±6.76	119.00±6.19	.757	.452
180 min	118.80±6.21	118.13±6.45	.408	.685
6 hours	118.07±6.31	117.0±5.38	.705	.484
12 hours	117.13±5.50	115.47±5.14	1.21	.230
24 hours	115.80±5.05	113.73±4.78	1.63	.109

The diastolic blood pressure (DBP) was consistently higher in Group A than in Group B at all time points. Statistically significant differences were observed at 30 min (p=0.036), 180 min (p=0.033), 12 hours (p=0.027), and 24 hours (p=0.046). Several other intervals showed near-significant trends. Overall, Group A maintained a higher DBP profile compared to Group B over 24 hours (Table 5). Table 6 shows that the oxygen saturation (SpO₂) levels remained comparable between Groups A and B at all time intervals, with no statistically significant differences (p > 0.05). Both groups maintained stable SpO₂ values close to 97-98% throughout the 24-hour period (Table 6).

**Table 5:** Comparison of diastolic blood pressure (mmHg) at different time intervals between Groups A and B Values are presented as mean ± standard deviation. DBP: diastolic blood pressure p-values were calculated using the Independent t-test. A p-value of <0.05 is considered as significant.

Diastolic Blood pressure(mmHg)	Groups		t value	p-value
	A	B		
Baseline	81.27±8.04	78.07±8.52	1.50	.140
0 min	81.13±7.92	78.07±8.52	1.44	.154
5 min	81.27±7.89	77.87±8.39	1.62	.111
10 min	80.93±7.59	77.53±8.03	1.69	.097
15 min	80.87±7.38	77.13±7.67	1.92	.060
30 min	80.53±7.12	76.47±7.51	2.15	.036
60 min	79.93±7.13	76.33±7.54	1.90	.062
90 min	78.67±6.69	75.47±7.82	1.70	.094
120 min	78.13±6.45	74.73±7.78	1.84	.071
150 min	78.00±6.35	74.47±7.71	1.94	.058
180 min	77.20±6.49	73.20±7.64	2.19	.033
6 hours	76.60±6.22	72.93±7.93	1.99	.051
12 hours	76.47±6.66	72.33±7.45	2.27	.027
24 hours	75.47±7.29	71.60±7.36	2.04	.046

**Table 6:** Comparison of oxygen saturation (SpO2, %) at different time intervals between Groups A and B Values are presented as mean ± standard deviation. SpO₂: peripheral capillary oxygen saturation p-values were calculated using the independent t-test. A p-value of <0.05 is considered significant.

Spo2 (%)	Groups		t value	p-value
	A	B		
Baseline	98.43±1.16	98.53±1.07	-.346	.731
0 min	98.47±1.17	98.43±1.01	.119	.906
5 min	98.23±1.07	98.23±.858	.000	1.00
10 min	98.03±.858	98.07±.944	-.136	.892
15 min	97.80±.925	97.87±1.01	-.267	.790
30 min	97.73±.944	97.60±1.10	.503	.617
60 min	97.57±1.16	97.37±1.07	.694	.491
90 min	97.33±1.18	97.27±.944	.241	.810
120 min	97.23±1.14	97.13±1.01	.361	.720
150 min	97.27±1.17	97.10±1.03	.585	.561
180 min	97.10±1.16	97.00±.947	.367	.715
6 hours	97.10±1.13	96.90±.960	.741	.462
12 hours	97.07±1.14	96.90±.922	.621	.537
24 hours	97.33±1.35	97.10±1.27	.690	.493

**Figure 2:**
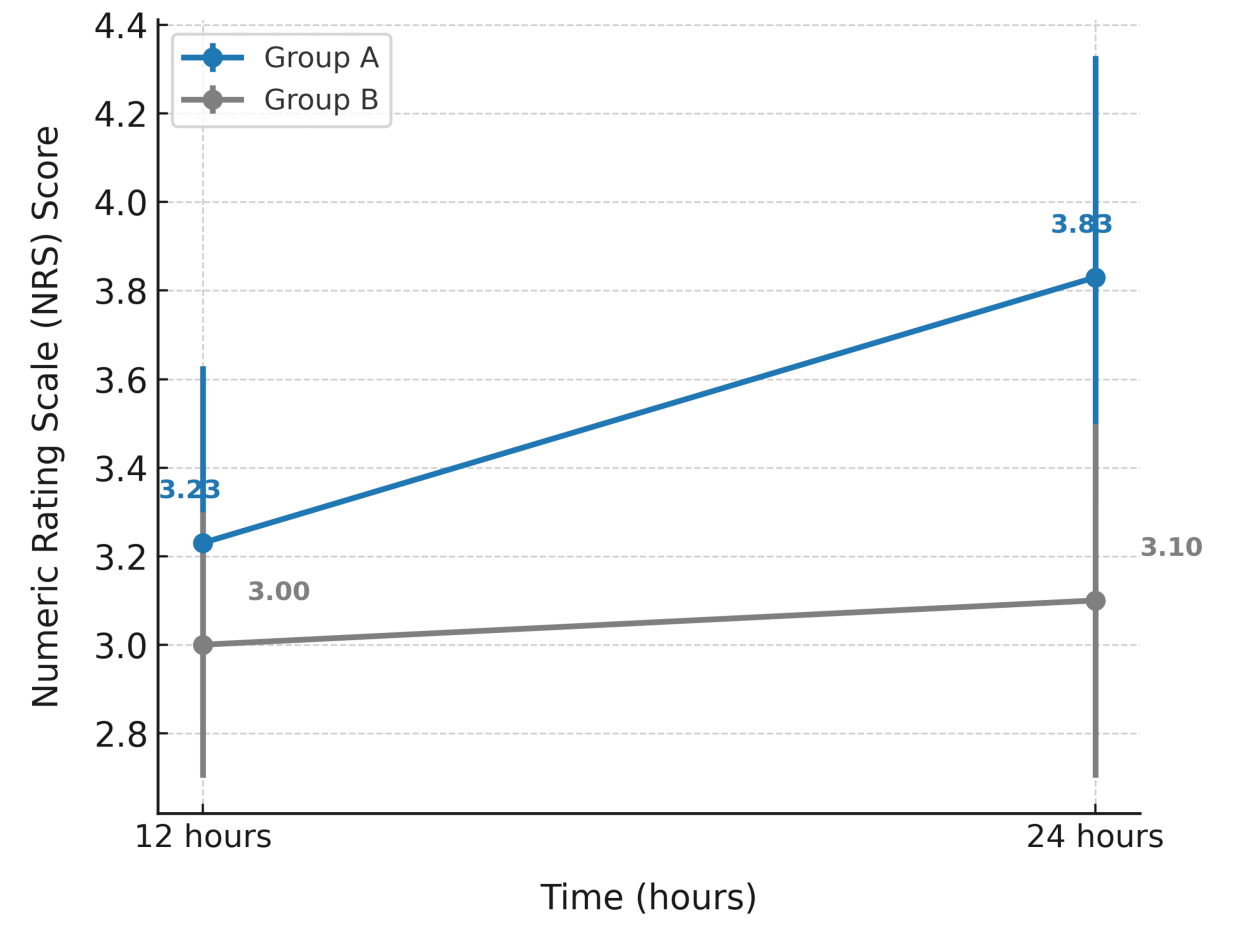
Comparison of NRS at different time intervals between groups NRS: numeric rating scale

A comparison of NRS scores at 12 and 24 hours between Groups A and B. Group A exhibited a noticeable increase in NRS scores from 3.23 at 12 hours to 3.83 at 24 hours, indicating a rise in perceived pain over time. In contrast, Group B maintained relatively stable scores, increasing only slightly from 3.00 to 3.10. This suggests that Group B experienced better pain control over the 24-hour period compared to Group A (Figure 2).

No cases of nausea, vomiting, tachycardia, bradycardia, hypotension, hypertension, or respiratory depression were observed in either group. This indicates that both interventions were well-tolerated, with no reported adverse events or hemodynamic instability in either group.

In our study, Group B demonstrated significantly prolonged sensory (866.33±82.73 vs. 405.33±39.28 min) and motor block durations (1060.00±96.13 vs. 381.67±38.24 min), extended analgesia duration (1171.67±96.74 vs. 470.0±46.24 min), and lower postoperative pain scores at 12 and 24 hours compared to Group A, with p = 0.001 in all parameters. These findings suggest the enhanced efficacy of the intervention used in Group B in terms of nerve blockade and postoperative analgesia, without compromising safety.

## Discussion

Ultrasound-guided supraclavicular brachial plexus block is a preferred regional anesthesia technique for upper limb surgeries because it provides dense anesthesia with rapid onset and prolonged postoperative analgesia. To further enhance block duration and quality, various adjuvants, such as α-2 agonists, opioids, and corticosteroids, have been explored, with dexamethasone emerging as one of the most effective options. In the present prospective, randomized, double-blind study comparing intravenous and perineural dexamethasone as adjuvants to levobupivacaine, we observed that perineural administration significantly shortened the onset and markedly prolonged the duration of both sensory and motor block. It also extended postoperative analgesia and maintained lower pain scores at 12 and 24 hours without compromising hemodynamic stability or increasing adverse effects. These results clearly establish the superiority of perineural dexamethasone over intravenous administration in optimizing block characteristics and postoperative pain control.

The sensory and motor block durations were shorter than those reported by Veena G et al. (2021) [25], possibly due to variations in drug dosage, adjuvant use, or patient characteristics. In contrast, our findings showed a stronger effect than Bisui B et al. (2017) [4], who reported shorter block durations, likely due to different anesthetic agents or the absence of adjuvants. Rao MV Kameswar et al. (2019) [26] also reported similar prolongation with adjuvant use, aligning with our findings.

The prolonged analgesia in Group B is supported by Veena G et al. [25], Rao MV Kameswar et al. [26], and further corroborated by Baeriswyl M et al. (2017) [27] and Chong A M et al. (2017) [5], who demonstrated the efficacy of perineural dexamethasone in prolonging pain relief. Our lower pain scores at 12 and 24 hours are consistent with Veena G et al. [25] and Vasconcelos M M et al. (2020) [28], highlighting sustained analgesia and reduced need for rescue medication in Group B.

Hemodynamic parameters remained stable across groups, differing from Prathibhan S et al. (2024) [29] and Parthasarathy P et al. (2018) [6], possibly due to differences in monitoring protocols or anesthetic regimens. Importantly, no significant complications were observed, confirming the safety of the Group B intervention, consistent with findings from Baeriswyl M et al. [27] and Chong A M et al. [5].

Our study's strengths include well-balanced baseline characteristics, comprehensive assessment of anesthesia parameters, objective pain scoring, and consistent findings with previous studies [4,5,25-27]. Overall, the results provide strong evidence supporting the efficacy and safety of the Group B intervention for enhanced postoperative pain management.

Limitations

The study was limited by a relatively small sample size and the single-center design, which may restrict generalizability. Only short-term postoperative outcomes were assessed, without evaluation of long-term analgesic efficacy. Subjective variation in pain perception and operator-dependent factors in performing ultrasound-guided blocks could also have influenced the results.

## Conclusions

Both intravenous and perineural dexamethasone effectively enhanced the quality and duration of ultrasound-guided supraclavicular brachial plexus blocks when combined with levobupivacaine. However, perineural dexamethasone resulted in a significantly slower onset and longer duration of sensory and motor block, as well as prolonged postoperative analgesia. Hemodynamic stability was maintained in both groups, and no major complications were observed. Perineural administration of dexamethasone is recommended as the preferred adjuvant in supraclavicular blocks to maximize block effectiveness and analgesia duration. Further studies with larger sample sizes and longer follow-up are suggested to confirm safety and explore the potential of combining dexamethasone with other adjuvants for enhanced perioperative pain control.
